# Innovation in Radionuclide Therapy for the Treatment of Prostate Cancers: Radiochemical Perspective and Recent Therapeutic Practices

**DOI:** 10.3390/cancers15123133

**Published:** 2023-06-10

**Authors:** Emmanuel Deshayes, Cyril Fersing, Constance Thibault, Mathieu Roumiguie, Philippe Pourquier, Nadine Houédé

**Affiliations:** 1 INSERM U1194, Montpellier Cancer Research Institute, University of Montpellier, 34298 Montpellier, France; 2 Department of Nuclear Medicine, Institute du Cancer de Montpellier (ICM), 34298 Montpellier, France; 3 IBMM, University Montpellier, CNRS, ENSCM, 34293 Montpellier, France; 4 Department of Medical Oncology, Hôpital Européen Georges Pompidou, Institut du Cancer Paris CARPEM, AP-HP Centre, 75015 Paris, France; 5 Urology Department, Andrology and Renal Transplantation, CHU Rangueil, 31059 Toulouse, France; 6 Medical Oncology Department, Institute de Cancérologie du Gard—CHU Caremeau, 30009 Nîmes, France

**Keywords:** prostate cancer, radionuclide therapy, lutetium-177, radium-223, castration resistance, PSMA

## Abstract

**Simple Summary:**

Despite the use of surgery and treatments that inhibit the androgen axis signaling, prostate cancer patients invariably become resistant to castration. In the case of metastatic disease, several alternatives have emerged in the past decade with the development of radionuclide therapies. These treatments may represent an interesting option as they could target either the microenvironment of sclerotic bone metastases or proteins that are specifically expressed at the surface of cancer cells, as it is the case with the prostate-specific membrane antigen. This review summarizes the clinical trials evaluating the efficacy of radionuclide therapies in patients with locally advanced or metastatic prostate tumors. Besides the theragnostic use of radionuclides, it also discusses the recent encouraging results that were obtained with ^177^Lu-PSMA-617 and the specific requirements for the use of this class of medication.

**Abstract:**

Prostate cancer represents the second cause of death by cancer in males in western countries. While early-stage diseases are accessible to surgery and/or external radiotherapy, advanced metastatic prostate cancers are primarily treated with androgen deprivation therapy, to which new generation androgen receptor antagonists or taxane-based chemotherapies are added in the case of tumor relapse. Nevertheless, patients become invariably resistant to castration with a median survival that rarely exceeds 3 years. This fostered the search for alternative strategies, independent of the androgen receptor signaling pathway. In this line, radionuclide therapies may represent an interesting option as they could target either the microenvironment of sclerotic bone metastases with the use of radiopharmaceuticals containing samarium-153, strontium-89 or radium-223 or tumor cells expressing the prostate-specific membrane antigen (PSMA), a protein found at the surface of prostate cancer cells. This review gives highlights the chemical properties of radioligands targeting prostate cancer cells and recapitulates the clinical trials evaluating the efficacy of radionuclide therapies, alone or in combination with other approved treatments, in patients with castration-resistant prostate tumors. It discusses some of the encouraging results obtained, especially the benefit on overall survival that was reported with [^177^Lu]-PSMA-617. It also addresses the specific requirements for the use of this particular class of drugs, both in terms of medical staff coordination and adapted infrastructures for efficient radioprotection.

## 1. Introduction

Prostate cancer is a major healthcare issue in industrialized countries as it represents the second cause of death by cancer in males, after lung and bronchus cancers [1]. All patients with metastatic disease now have access to a combination treatment associating androgen deprivation therapy (ADT, e.g., goserelin, leuprorelin or triptorelin) with either docetaxel, abiraterone acetate or androgen receptor (AR) antagonists such as enzalutamide, apalutamide or darolutamide [2,3]. Nevertheless, patients eventually become resistant to castration and, despite the use of taxane-based chemotherapies, the median survival of metastatic castration-resistant prostate cancer (mCRPC) patients seldom exceeds 3 years [4]. Hence, there is an unmet need for therapeutic alternatives independent of the androgen receptor signaling pathway. Radiolabeled drugs appeared as a promising therapeutic option, targeting either the microenvironment of sclerotic bone metastases (e.g., samarium-153 lexidronam [5], strontium-89 dichloride [6] or radium-223 dichloride [7]) or, more recently, directly tumor cells (e.g., PSMA ligands labelled with lutetium-177). ^177^Lu-PSMA radiopharmaceuticals specifically bind to prostate-specific membrane antigen (PSMA), a target protein that is overexpressed at the surface of prostate cancer cells [8]. The aim of this review is to present the different radionuclide therapies available and their positioning in the current strategies for prostate cancer treatment. We will also review the current ongoing clinical trials involving such radiopharmaceuticals and discuss the practical issues related to the use of these radionuclide-containing drugs.

## 2. Radionuclide Therapy

In nuclear medicine, radiolabeling of specific probes with either gamma-(^99m^Tc, ^111^In, etc.) or positron-emitting radioisotopes (^18^F, ^68^Ga, etc.) allows non-invasive visualization of molecular targets at the whole-body level with scintigraphy or PET/CT devices. When targets are sufficiently expressed, the same probes may be radiolabeled with particle-emitting (i.e., beta minus or alpha) radioisotopes, delivering higher energy to eradicate tumor cells. This strategy is referred to as a theragnostic approach [9]. The ideal situation would require that the target is specifically expressed in tumor cells and that the probe would selectively recognize this target while its unbound fraction would be rapidly cleared from the blood. Radionuclides are usually bound to the vectors via a chelating agent, and vectors are, in most cases, the same for diagnostic and therapeutic purposes. However they could sometimes differ due to physicochemical stress or drug development limitations; in these cases, the final target remains the same.

**Figure 1:**
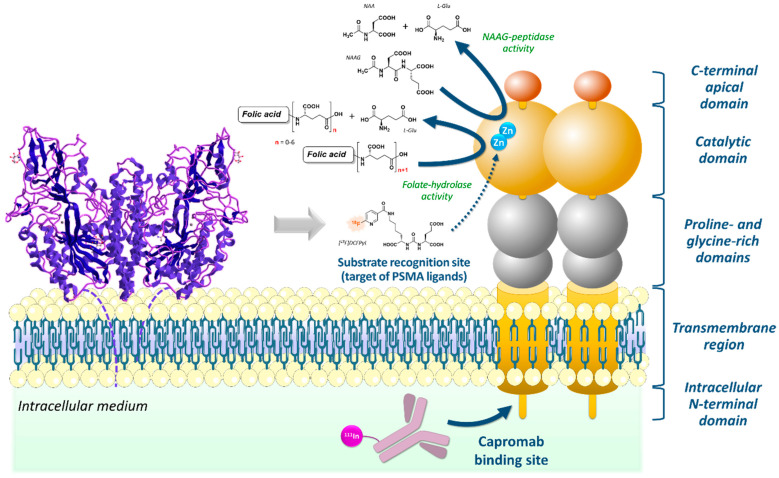
Crystal structure of PSMA in its noncovalent homodimeric form; PDB: 1Z8L (left) [16] and schematic representation of the PSMA homodimer. The full-length monomeric protein (~100 kDa molecular weight) includes a transmembrane region (24 amino acids), a short N-terminal cytoplasmic sequence (19 AA) which contains the capromab binding site, and a large, highly glycosylated extracellular domain (707 AA, 9 predicted N-glycosylation sites). The extracellular domain contains two domains of unknown function (106 and 123 AA) separated by proline- and glycine-rich regions (27 and 24 AA) and an exopeptidase-like catalytic domain containing the PSMA ligands recognition site. Additionally, a helical dimerization domain is located at the C-terminal end, with no known functions. PSMA that is expressed in central nervous system especially metabolizes the brain neurotransmitter N-acetyl aspartyl glutamate (NAAG) and is named NAALADase. In the proximal small intestine, it removes γ-linked glutamates from polyglutamated folate and is named folate-hydrolase (FOLH1). Both enzymatic reactions produce *L*-glutamate and are catalyzed within a binuclear zinc active site, which is also the recognition site of urea-based PSMA ligands [17,18]. Only the dimeric form of PSMA has enzymatic activity and is expressed on the surface of prostate cells [19].

PSMA, also known as folate-hydrolase I, is a transmembrane glycoprotein with a glutamate-carboxypeptidase enzymatic activity (Figure 1). It is particularly overexpressed in prostate cancer cells, especially in metastatic or advanced stages. Despite its name, PSMA is not specific to prostate cancer and may be expressed in normal prostate tissue, in several other organs (e.g., lacrimal and salivary glands, liver, spleen, kidney, brain, duodenum) or in other tumor types [10]. Several radiolabeled probes directed against PSMA have been designed for imaging and therapy over the last decades. The first drug to be marketed was a conjugated murine monoclonal antibody, capromab pendetide (CYT-356, Prostascint^®^, EUSA Pharma, Hemel Hempstead, UK), radiolabeled with ^111^In to perform scintigraphy imaging [11,12]. Because it was directed against an epitope in the intracellular domain of PSMA, it resulted in low sensitivity. Indeed, only non-viable cells with membrane impairment and exposure of the intracellular domain of PSMA may be targeted (i.e., necrosis). Another drawback of this radiolabeled antibody was the long delay between injection of the radiotracer and imaging (4 to 5 days) to allow sufficient clearance of the blood compartment and increase contrast. It led to the development of low molecular weight inhibitors of PSMA targeting the extracellular part of the glycoprotein (Figure 1). These molecules, mostly based on the glutamate-urea-lysine (KuE) chemical scaffold, usually have a fast blood clearance. Because the binding of PSMA ligands to their target induces internalization [13], their intracellular retention makes them ideal for theragnostic applications, as presented in details in recent reviews [14,15].

**Figure 2:**
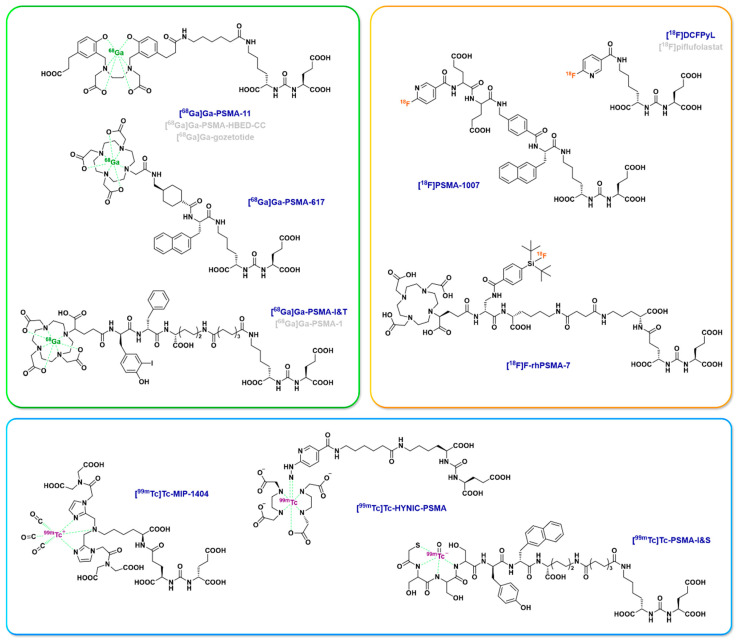
Chemical structure of selected PSMA ligands for ^68^Ga PET imaging (green box), ^18^F PET imaging (orange box) and ^99m^Tc SPECT imaging (blue box). Each of these compounds is based on the targeting motif “glutamate-urea-R”, with R = lysine or glutamate. As the PSMA active site is specific for C-terminal glutamate residues, the glutamate part of PSMA ligands is used for target binding. The urea motif is useful for mimicking a peptide bond while preventing the ligand from being a PSMA substrate. The R part will be used for the connection of the radioactive group, possibly with a linker chain. Concerning the most commonly used ligand in ^68^Ga imaging, PSMA-11, its HBED-CC chelator allows easy radiolabeling at room temperature but is not compatible with other radiometals, whereas the DOTA chelator of PSMA-617 or PSMA-I&T is much more versatile and can also form stable complexes with radioelements for therapy (e.g., ^177^Lu, ^90^Y, ^225^Ac). Regarding ^18^F-containing molecules, DCFPyL and PSMA-1007 are radiolabeled through the substitution of the KuE motive by the [^18^F]-6-fluoronicotinic acid prosthetic group. In contrast, rhPSMA-7 is a radiohybrid PSMA ligand [35] for which fluorination is based on an isotope exchange approach. Lastly, the chelating groups of the PSMA ligands for SPECT imaging are very specific for this radiometal, such as a carboxylic acid-substituted bis-imidazole moiety in MIP1404, a hydrazinonicotinamide and di-ethylenediaminediacetic acid association in HYNIC-PSMA or a mercaptoacetyl triserine in PSMA-I&S.

It has been only 10 years since the first report of a patient imaged with a ^68^Ga-labeled PSMA ligand (PSMA-HBED-CC or PSMA-11 or gozetozide) using PET/CT [20]. In the meantime, other PSMA ligands for PET imaging have been studied such as [^68^Ga]Ga-PSMA-617, [^68^Ga]Ga-PSMA-I&T (PSMA-1 or PNT-2002), [^18^F]DCFPyL ([^18^F]piflufolastat), [^18^F]PSMA-1007, [^18^F]rhPSMA-7 or [^68^Ga]Ga-rhPSMA-7, with advantages and disadvantages related primarily to the physical and chemical properties of the radioisotope [21]. Additionally, the nature of the vector itself can have a direct impact on its accumulation in the urinary tract that is low for PSMA-1007 and PSMA-617 (which is of essential interest to reduce kidney irradiation, especially with [^177^Lu]Lu-PSMA-617) but gets higher with PSMA-11 (that can complicate the identification of recurrences in the prostate or pelvic lymph nodes on PET imaging) [22]. This may be partly due to the nature of the chelating agent, as a lower renal uptake has been observed for DOTA-containing PSMA derivatives than for HBED-CC-containing analogs [23,24]. Additionally, the use of vectors labelled with gamma-emitting radioisotopes to perform scintigraphy imaging, such as [^99m^Tc]Tc-MIP-1404 ([^99m^Tc]Tc-Trofolastat) [25], [^99m^Tc]Tc-PSMA-I&S [26] or [^99m^Tc]Tc-HYNIC-PSMA [27], has also been reported. Figure 2 summarizes the main PSMA ligands used in imaging, some of which have dual use in both imaging and therapy. Radioligand therapy (RLT) targeting PSMA was first introduced with ^131^I-MIP-1095 [28,29], followed by an increasing number of studies using ^177^Lu-labeled PSMA ligands (clinical studies will be detailed below). ^177^Lu is a radiometal with advantageous physicochemical properties, displaying a short tissue penetration (2–3 mm) and a relatively long half-life (6.7 days). Moreover, it can form stable complexes, especially with chelators derived from 1,4,7,10-tetraazacyclododecane-1,4,7,10-tetraacetic acid (DOTA). This allows ^177^Lu-PSMA-based radiopharmaceuticals to be produced either in-house [30,31,32,33] or, more frequently, by industrial radiopharmaceuticals manufacturers [34].

**Figure 3:**
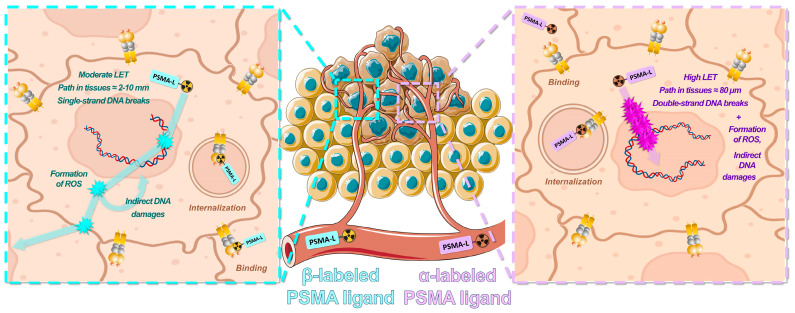
Radiobiology principles associated with PSMA RLT using β-labeled (**left**) or α-labeled (**right**) PSMA ligands.

In radiation therapy, tumor cell death is directly related to the absorbed doses (energy deposit expressed in Grays, with 1 Gy = 1 J/kg) through damages to DNA that may be direct or indirect (water ionization or excitation, generating reactive oxygen species) following interaction with the ionizing particle or radiation. In comparison with external beam radiation therapy (EBRT), RLT has some specificities. The radiation dose distribution is usually heterogeneous in the whole body and in tissues, with a protracted exposure and low absorbed dose rate irradiations. Contrary to photons and electrons that are produced in sealed sources for EBRT, RLT is associated with the emission of beta minus (e.g., for ^131^I or ^177^Lu) or alpha (e.g., for ^211^At or ^225^Ac) particles that are produced by the radioisotope linked to the vector. In EBRT, dosimetry is defined before treatment whereas it is usually not calculated before nor during treatment in the case of RLT. Indeed, to assess the dose values deposited in organs or tumors following RLT, one has to take into account the pharmacokinetics of the radiolabeled drug, which may vary between each patient (cumulated activities) [36,37,38,39]. It can be assessed by performing post-therapeutic gamma radiation imaging at various time-points post-injection when the radioisotope displays a co-emission of gamma particle, as is the case for ^177^Lu or ^131^I [15]. Moreover, for the same absorbed dose, all types of radioactive emitters do not have the same biological effects. For instance, alpha-particle emitters present with a high linear energy transfer (LET), which represents the energy deposit by length (or volume). Compared to beta-particle emitters and for the same physical absorbed dose, these isotopes will generate higher density of ionization and excitation along their track, leading to various types of damages which are more difficult to repair (DNA single-strand and double-strand breaks, as well as base modifications) (Figure 3) [40,41]. Therefore, clinical PSMA RLT applications with alpha-emitters such as ^225^Ac (t_1/2_ = 9.9 days) may be useful as, based on current knowledge, the heavy cellular damages caused by short-range alpha particles are not associated with known mechanisms of resistance [42,43,44].

**Figure 4:**
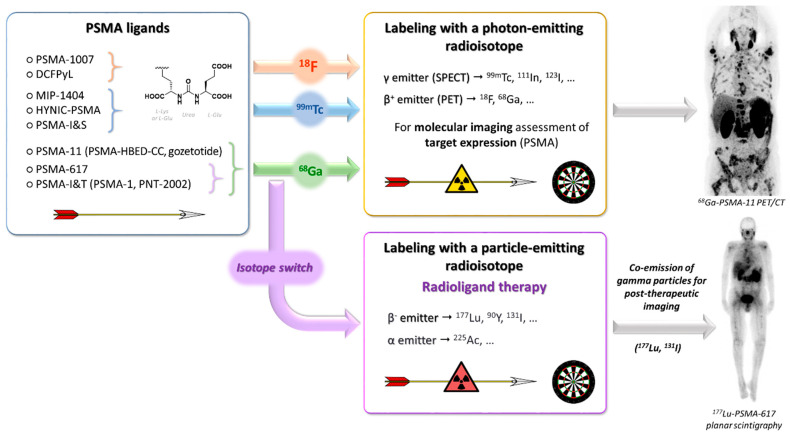
Theragnostic approach applied to the management of prostate cancer by targeting PSMA: from imaging to therapeutic activity.

Most clinical studies on PSMA RLT use ^177^Lu-labeled PSMA ligands. They are performed with fixed injected activities of the radioactive drug (expressed in Bq), administered to the patients at fixed intervals without considering dosimetry on lesions or on healthy organs. The dosimetry calculations require specific procedures [45] and may help in the selection of patients and to adapt the treatment schedules or individualize the amount of injected activity. Recently, a simplified 3D dosimetry approach following ^177^Lu-labeled PSMA RLT showed a tumor dose–response relationship with a significant correlation between whole-body tumor-absorbed dose and serum prostate-specific antigen (PSA) decline. The patients who received less than 10 Gy were unlikely to achieve a decline in PSA levels of more than 50%. In this study, injected activities were adjusted for patients’ weight, renal function and tumor burden (the injected activity was increased as a function of number of tumor sites to take into consideration the “tumor sink effect”) [46]. Figure 4 schematizes the theragnostic concept applied to PSMA targeting and recapitulates the main radioelements that can be used in this approach. 

## 3. Results of Clinical Trials Involving Radionuclide Therapy in Prostate Cancers

### 3.1. Radium-223

Several radionuclide therapies have been evaluated in metastatic prostate cancer. Phosphorus-32 [47], strontium-89 dichloride (Metastron™, Q BioMed, New York, NU, USA) [6,48] and samarium-153 lexidronam (Quadramet^®^, Lantheus Medical Imaging, Billerica, MA, USA) [5,49] are the oldest. Nevertheless, Metastron™ and Quadramet^®^ are still in use. They were efficient to relieve cancer-related bone pain but failed to improve progression-free survival (PFS) and overall survival (OS) when used as monotherapy (probably due to insufficient tumor cell control with beta emitters that may be overcome by alpha emitters). Radium-223 dichloride (Xofigo^®^, Bayer, Leverkusen, Germany) was the first radionuclide therapy to show an overall survival benefit in patients with mCRPC (ALSYMPCA clinical trial, 14.9 months vs. 11.3 months, hazard ratio (HR) = 0.7, 95% CI 0.58–0.83, *p* < 0.001) [7]. It was approved by FDA and EMA in 2013. This α-emitting radionuclide that belongs to the alkaline earth metal group, like calcium and strontium, concentrates on the bone surface due to its affinity for osteoblasts and, therefore, specifically targets bone metastases. However, inclusions in the ALSYMPCA clinical trial were limited to patients with symptomatic bone metastases in the absence of visceral disease. Moreover, half of the patients enrolled in this clinical trial were docetaxel-naïve because they were too frail or refused the chemotherapy. Of note, none of the patients had been previously treated with androgen receptor pathway inhibitor (ARPI) or with cabazitaxel because those treatments were not available at that time. Therefore, subsequent clinical trials have been conducted to evaluate the efficacy and safety of ARPI + radium-223 combination therapy, among which the EORTC 1333/PEACE 3 trial with enzalutamide (NCT02194842) and the ERA 223 trial with abiraterone acetate (NCT02043678). The ERA 223 trial was terminated prematurely due to excessive rate of skeletal-related events [50]. This highlighted the importance of using bone-protecting agents (BPAs) in association with radium-223, as in both the intervention and control arms, frequency of fractures was lower when patients received BPAs with their assigned therapy. Since then, an amendment was made in PEACE 3 to compel the use of a BPA such as the anti-receptor activator of nuclear factor-kappa B ligand (RANK-L) antibody denosumab. To date, the enrollment of this study is still ongoing. Currently, the EMA recommends restricting the use of radium-223 alone. Moreover, it is limited to patients with bone metastatic prostate cancer who have had two previous treatments or who cannot receive other treatments. Nonetheless, its clinical use is expected to increase due to its reimbursement in a growing number of countries. 

### 3.2. Radiolabeled PSMA Ligands

More recently, promising results have been reported with ^177^Lu-PSMA ligands. Contrary to radium-223, which is an alpha-emitting radioelement with a natural tropism for the bone, ^177^Lu-PSMA radiopharmaceuticals is a β-emitting radionuclide carried by a pseudo-dipeptide drug that binds to PSMA. One single arm phase II clinical trial (LuPSMA, ACTRN12615000912583) evaluated [^177^Lu]Lu-PSMA-617 in mCRPC patients previously treated by taxane-based chemotherapy and ARPI [51]. The treatment consisted in four cycles of intravenous [^177^Lu]Lu-PSMA-617 (6 GBq per cycle adjusted to tumor burden, patient weight and renal function) every 6 weeks. The primary endpoint was PSA response, defined as a greater than 50% PSA decline from baseline. Among the 43 patients enrolled, 40% had previously received one chemotherapy regimen, and 40% had previously received two regimens. All patients had two PET/CT: one [^68^Ga]Ga-PSMA-11 PET imaging to confirm the high PSMA expression and one [^18^F]FDG PET imaging to exclude patients with PSMA-negative FDG-positive lesions. A total of 43 patients were screened, and 30 patients were included. Half of the patients had a PSA response (57%) and 70% had a PSA decline of at least 30%. Among the 17 patients with measurable disease, the objective response rate was 82% (*n* = 14).

In a randomized phase II clinical trial (TheraP, NCT03392428), [^177^Lu]Lu-PSMA-617 was compared to cabazitaxel in patients previously treated with docetaxel and at least one ARPI [52]. As in the previous phase II trial, [^68^Ga]Ga-PSMA-11 and [^18^F]FDG PET/CT were performed before randomization with the same inclusion criteria. [^177^Lu]Lu-PSMA-617 was given at the dosage of 6.0–8.5 GBq intravenously every 6 weeks for up to six cycles, and cabazitaxel was administered at 20 mg/m^2^ every 3 weeks for up to ten cycles. The primary endpoint was PSA response; PFS, OS and safety profile were secondary endpoints. In total, 291 patients were screened, and 200 were randomized. [^177^Lu]Lu-PSMA-617 appeared superior to cabazitaxel in terms of PSA response (66% vs. 37%, *p* < 0.0001) and PFS (HR = 0.63, 95% CI 0.46–0.86, *p* = 0.0028). However, the median OS was similar in both arms (19.1 months in the [^177^Lu]Lu-PSMA-617 arm vs. 19.6 months in the cabazitaxel arm, 95% CI -3.7–2.7) [53]. Of note, 20 patients in the cabazitaxel arm received [^177^Lu]Lu-PSMA-617 as subsequent treatment.

The VISION clinical trial is the first phase III randomized study that compared standard of care (SoC) alone or in combination with [^177^Lu]Lu-PSMA-617 in mCRPC patients previously treated with at least ARPI and one or two taxane regimens [54]. SoC could be supportive care, ARPI, corticoid, radium-223 or experimental drugs but not chemotherapy. Patient selection was based on [^68^Ga]Ga-PSMA-11 PET/CT only. Further radiological assessment consisted in CT scan or MRI and bone scan every 8 weeks during 6 months, then every 12 weeks. Patients were randomized at a 2:1 ratio between [^177^Lu]Lu-PSMA-617 (7.4 GBq every 6 weeks for 4–6 cycles) plus SoC and SoC alone. The treatment was programmed for a total of four cycles, but patients with measurable responses, signs of residual disease and good tolerance could receive two additional cycles. The primary endpoint was OS. A total of 831 patients were included after the screening of 1179 patients. A majority of them (65%) had received only one prior taxane regimen, and half of them (55%) received concomitant ARPI as part of SoC. A benefit was observed in favor of [^177^Lu]Lu-PSMA-617 in terms of radiological PFS (median, 8.7 vs. 3.4 months; HR = 0.40; 99.2% CI 0.29–0.57; *p* < 0.001) and OS (median, 15.3 vs. 11.3 months; HR = 0.62, 95% CI 0.52–0.74; *p* < 0.001). The median number of cycles of [^177^Lu]Lu-PSMA-617 was 5 (1–6). The benefit was consistent regardless of the number of prior ARPI or taxane regimens and regardless of the concomitant treatments [55]. Of note, the OS benefit was even greater when [^177^Lu]Lu-PSMA-617 was associated with ARPI (HR = 0.55, 95% CI 0.43–0.70). Regarding tolerance, [^177^Lu]Lu-PSMA-617 had an acceptable safety profile. The most common adverse events observed in the VISION trial were fatigue (43%, including 6% of grade ≥ 3), dry mouth (39%, grade 1–2 only), nausea (35%), anemia (32%, including 13% of grade ≥ 3) and thrombocytopenia (17%, including 8% of grade ≥ 3). Only 6% of the patients required a dose reduction and 12% a discontinuation due to adverse events. 

## 4. Ongoing Clinical Trials Using Radionuclide Therapies in the Treatment of Prostate Cancers

Table 1 presents the ongoing phase 2 or 3 clinical trials using radionuclide therapies for the treatment of prostate cancers. For exhaustive survey, phase 1 studies are summarized in the Supplemental Table S1. Although the VISION study has already been published, confirming the position of [^177^Lu]Lu-PSMA-617 in metastatic hormone-refractory prostate cancers, several phase 2 and 3 clinical trials are currently conducted in different countries. In countries where radium-223 is available, phase 4 real life studies are also recruiting.

Based on the results of [^177^Lu]Lu-PSMA-617 in the advanced stages of the disease, many clinical trials are underway, either at earlier stages or in combination therapy. [^177^Lu]Lu-PSMA-617 is especially tested in oligometastatic hormone-sensitive prostate cancer (HSPC), associated with androgen deprivation in phase 2, and is compared to SoC (abiraterone acetate or enzalutamide) in phase 3 clinical trials. In oligometastatic HSPC, in which the objective is to control all metastatic sites, [^177^Lu]Lu-PSMA-617 is tested in combination with external beam radiation therapy or radiation stereotactic ablative radiotherapy. Sequential treatment is also investigated in phase 2 using [^177^Lu]Lu-PSMA-617 upfront for two cycles followed by six cycles of docetaxel, in order to reach a higher proportion of patients with a biological PSA response as compared to patients receiving docetaxel and ADT. 

Regarding the combination strategy of radionuclide therapy with other antitumor agents in the treatment of prostate cancer, the approach can be intended for the following:

- To improve the radiosensitivity of the tumor. It is known that androgens downregulate FOLH1 gene expression, which in turn reduces the level of PSMA mRNA levels and, consequently, PSMA expression. Conversely, ADT upregulates the FOLH1 gene and thereby increases PSMA expression in PSMA-low prostate cancer cell lines as shown in a patient’s case of mCRPC [56,57]. Another report also suggests that AR antagonists such as enzalutamide could enhance the expression of PSMA [58]. In the VISION clinical trial, patients were allowed to receive approved hormonal treatments (including abiraterone and enzalutamide) as SoC combined with [^177^Lu]Lu-PSMA-617, but only few of them received the combination, which made it difficult to evaluate clinical benefit of this association. A separate study published preliminary results in 10 patients with insufficient response to [^177^Lu]Lu-PSMA-617 who received, subsequently, enzalutamide, showing synergistic effects with a PSA decrease after at least two cycles of the association [59]. These encouraging results further emphasize the need to test such combination protocols involving oral targeted therapies in future clinical trials.
- To enhance the radiation-induced immunologic shift and increase the efficacy of immunotherapy. The emergence of immune checkpoint inhibitors has changed the landscape of treatment in many solid cancers [60]. However, the low immunogenicity of prostate cancers limits the benefit of treatments such as programmed death receptor 1 (PD-1) inhibitors. A phase 1b, single arm study (NCT03805594) investigated the potential immunogenic priming effect of a single dose of [^177^Lu]Lu-PSMA-617 in chemotherapy-naïve patients with mCRPC, subsequently treated with pembrolizumab (anti-PD1) [61]. Among the 18 patients enrolled, the median radiological PFS was 6.5 months (95% CI 2.5–9.8). Interestingly, somatic genomic data showed that all patients carried low tumor mutational burden. A case report also mentions two patients treated with ^177^Lu-labeled PSMA concomitantly with pembrolizumab and sequentially with Olaparib (anti-PARP oral therapy), respectively [62]. The first patient showed PSA stabilization after three cycles, and the second patient achieved radiological and biochemical response. More recently, the PRINCE phase I clinical trial (NCT03658447) recruited 37 mCRPC patients who received up to six cycles of [^177^Lu]Lu-PSMA-617 in conjunction with pembrolizumab for up to 2 years [63]. Median radiological PFS, PSA-PFS and OS were 11.2 months (95% CI 5.1–14.1), 8.2 months (95% CI 5.1–11.2) and 17.8 months (95% CI 13.4–not estimable). No new safety concerns were observed, encouraging further evaluation of this type of combination.

Notably, combination therapy associating PSMA RLT and cytotoxic chemotherapy within a same protocol are, to our knowledge, not yet studied, as is the case with DOTATATE in neuroendocrine tumors (e.g., CAAA601A42101 clinical trial, NCT05142696).

## 5. Clinical Management Associated with the Use of Radionuclides

Based on the results of the aforementioned VISION clinical trial, the FDA approved lutetium-177 vipivotide tetraxetan ([^177^Lu]Lu-PSMA-617) (Pluvicto™, Advanced Accelerator Applications, Novartis, Basel, Switzerland) on 23 March 2022 for the treatment of patients with PSMA-positive mCRPC who have undergone treatment with inhibitors of the androgen axis and taxane-based chemotherapy [64], followed by EMA authorization on 9 December 2022. PSMA-positive mCRPC patients are defined as having at least one tumor lesion with a [^68^Ga]Ga-PSMA-11 uptake greater than normal liver. As in the pivotal clinical trial, patients are not eligible for this treatment if any lesion exceeded a certain size in the short axis and had an uptake lower than or equal to normal liver [65].

Unfortunately, approximately 30% of patients do not respond to RLT with [^177^Lu]Lu-PSMA-617 [54], particularly because of a decrease in tumor expression of PSMA or a heterogeneous tumor expression of PSMA. In this context, Buteau et al. reported during ASCO-GU 2022 on [^68^Ga]Ga-PSMA-11 and [^18^F]FDG PET imaging as potential predictive and prognostic markers [66]. In men with mCRPC progressing after docetaxel in the TheraP/ANZUP 1603 clinical trial mentioned above (which evaluated [^177^Lu]Lu-PSMA-617 vs. cabazitaxel in mCRPC), SUVmean ≥ 10 in [^68^Ga]Ga-PSMA-11 imaging was predictive of a higher likelihood of favorable response to [^177^Lu]Lu-PSMA-617 than to cabazitaxel, whilst a high metabolic tumor burden on [^18^F]FDG PET (often found in PC with neuroendocrine features and possibly induced by glucose transporters overexpression [67]) was associated with a worse prognosis regardless of the randomly assigned treatment. Therefore, performing both [^68^Ga]Ga-PSMA-11 and [^18^F]FDG PET/CT may be helpful before deciding to treat with RLT in order to discriminate patients with heterogeneous disease and PSMA-negative lesions who are likely to be non-responsive. 

Although there is no formal contraindication for [^177^Lu]Lu-PSMA-617 treatment, its toxicity profile requires increased vigilance regarding the patient’s bone marrow reserve and general condition. Only 8% of patients in the VISION clinical trial had a PS2 status. Most common clinical adverse reactions (≥20%) are fatigue, dry mouth, nausea, anemia, decreased appetite and constipation. Most common laboratory abnormalities (≥30%) are lymphopenia, anemia, leukopenia, thrombocytopenia, hypocalcemia and hyponatremia [54]. There is no specific recommendation in the geriatric population, since 27% of the patients enrolled in VISION were 75 years or older. 

In practice, recommended dosage is 7.4 GBq (200 mCi) every 6 weeks. Patients should be monitored for toxicity every 3 weeks, including hematological surveillance, kidney function evaluation and adaptation of antalgic treatments if necessary. Since the drug is primarily eliminated by the kidney, patients should be advised to remain well hydrated and to urinate frequently in order to reduce bladder irradiation. 

Radiation exposure during and after treatment with [^177^Lu]Lu-PSMA-617 should also be minimized consistently with institutional good radiation safety practices and patient treatment procedures. Following FDA recommendations, management of adverse events may require temporary dose interruption (extending intervals between injections from every 6 weeks up to every 10 weeks), dose reduction or permanent discontinuation of the treatment. If a treatment delay due to an adverse reaction is required for more than 4 weeks, treatment with [^177^Lu]Lu-PSMA-617 must be discontinued. The dosage of [^177^Lu]Lu-PSMA-617 may be reduced by 20% to 5.9 GBq (160 mCi) and reassessed at the next treatment cycle for a possible reintroduction of the initial dosage if the toxicity is resolved. If a patient has further adverse reactions that would require an additional dose reduction, the treatment must be discontinued. 

Response to treatment must be clinically monitored every 6 weeks with pain evaluation and PSA testing. Optimal monitoring of treatment response by imaging is not clearly defined. In the VISION clinical trial, imaging was performed every 8 weeks, which is not consistent with the injection of [^177^Lu]Lu-PSMA-617 every 6 weeks. Expert opinion considers that restaging every 12 weeks, without toxicities or clinical progression, could be a suitable option [68]. With regard to the results of the VISION clinical trial, the maximum number of injections of [^177^Lu]Lu-PSMA-617 is six.

Furthermore, rechallenge therapy with ^177^Lu-PSMA in early responders has been shown to be safe and effective. This benefit was evidenced retrospectively in 30 patients who had received a median of three cycles of rechallenging therapy, with acceptable safety results [69]. 

## 6. Looking Ahead: The Near Future of Prostate Cancer Radionuclide Therapy

**Figure 5:**
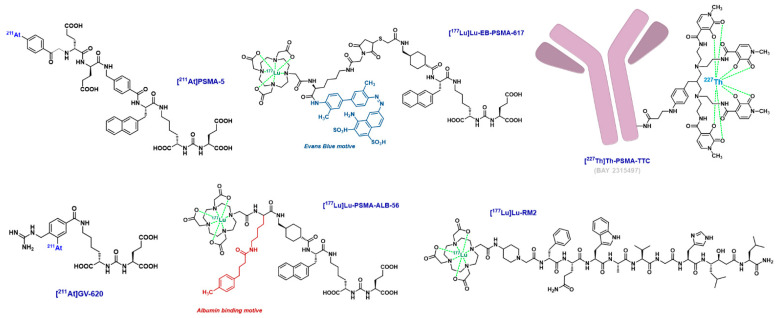
Chemical structures of selected prostate-targeting radionuclide therapy candidates.

Despite encouraging results with ^177^Lu-PSMA, some patients will not respond, and most of them will finally relapse. We detail in this section some perspectives to counterbalance it. In addition to the clinical trials mentioned above, the clinical and preclinical study of new therapeutic radioisotopes (i.e., alpha emitters), new vector molecules targeting PSMA or other targets will point to future breakthroughs in prostate cancer radionuclide therapy. Figure 5 illustrates some of these radiopharmaceuticals in development.

As it has already been studied in several patient cohorts, targeted alpha therapy involving ^225^Ac-labeled PSMA ligands will probably be one of the most impending and notable advances in the management of mCRPC patients. Initially reported in 2016 in two patients in a clinically critical situation, [^225^Ac]Ac-PSMA-617 at a rate of 3–4 cycles of 100 kBq/kg every 2 months showed impressive biological and radiological responses [70]. The same group confirmed these promising results within a cohort of 35 patients (median duration of tumor control under [^225^Ac]Ac-PSMA-617 last-line therapy = 9.0 months; response > 2 years for 5 patients) [71]. [^225^Ac]Ac-PSMA-I&T has also been studied in humans [72] and provided very comparable efficacy results as compared to ^255^Ac-labeled PSMA-617 [73,74]. More than with ^177^Lu-labeled PSMA ligands, xerostomia was the most common adverse effect in the cohorts studied [75,76,77,78] and sometimes required activity adjustment to improve tolerance [79,80,81]. Interestingly, a [^225^Ac]Ac-PSMA-617/[^177^Lu]Lu-PSMA-617 tandem therapy could also allow a successful response to radionuclide therapy while minimizing xerostomia severity [82,83,84]. Hematological toxicities remain uncommon with this treatment, especially in the absence of risk factors [85]. Overall, as with actinium-radiolabeled somatostatin analogs in the treatment of neuroendocrine tumors, the promising early results of targeted alpha therapy with PSMA ligands suggest that the conclusions of prospective randomized trials comparing [^225^Ac]Ac-PSMA-617 or PSMA-I&T with current standard treatment options will be of great clinical importance.

Ligands of PSMA radiolabeled with other alpha particle-emitting radioelements have also been designed and studied at the preclinical stage, some of which may potentially continue their development into the clinic. This may include ^211^At-labeled molecules, either analogs of DCFPyL [86,87], PSMA-1007 derivatives [88] or original pharmacokinetically optimized agents [89]. Only a few reports mention ligands of PSMA radiolabeled with bismuth-213 [90,91,92,93], this radioelement being difficult to handle in view of its very short physical half-life of 45.6 min. A *p*-SCN-Bn-DOTAM derivative of PSMA-617 radiolabeled with lead-213, ^212^Pb-NG001, has also been described [94,95,96,97], as well as PSMA ligands radiolabeled with terbium radioisotopes for theragnostic applications [98,99,100]. Interestingly, a ^227^Th-labeled PSMA-specific monoclonal antibody (Th-PSMA-TTC, BAY2315497) bearing a 3,2-HOPO chelator moiety is currently in phase 1 clinical trial to evaluate its safety, tolerability, pharmacokinetics and antitumor activity, alone or in combination with darolutamide, in patients with mCRPC (NCT03724747) [101,102,103].

Analogs of previously described vectors, such as bivalent molecules co-targeting albumin, displaying increased tumor uptake because of their enhanced blood circulation properties, were recently evaluated in first-in-human studies. [^177^Lu]Lu-PSMA-ALB-56 was injected in 10 patients with mCRPC (3360 ± 393 MBq) and showed normalized absorbed doses up to 2.3-fold higher in tumor lesions (6.64 ± 6.92 Gy/GBq) than conventional ^177^Lu-labeled PSMA ligands. Doses were similar in salivary glands (0.87 ± 0.43 Gy/GBq). However, kidneys and red marrow were more exposed to radiation (2.54 ± 0.94 Gy/GBq and 0.29 ± 0.07 Gy/GBq, respectively) [104]. Similarly, [^177^Lu]Lu-EB-PSMA-617 displayed comparable early results and also proved to be effective, with response correlated with treatment dose (up to 2.12 GBq) [105,106,107]. This radionuclide therapy is currently being investigated in two phase 1 clinical trials involving mCRPC patients (NCT03403595, NCT03780075). Lastly, ibuprofen-containing derivatives such as [^177^Lu]Lu-Ibu-DAB-PSMA are currently evaluated at the preclinical stage and are characterized by a more favorable safety profile as compared to [^177^Lu]Lu-PSMA-ALB-56, especially considering hematological toxicity [108,109,110,111].

Beyond the small molecule ligands of PSMA, other targeting agents remain good candidates for prostate cancer radionuclide therapy. As such, monoclonal antibodies targeting extracellular epitopes of PSMA are given special attention. The construct [^177^Lu]Lu-rosapatumab (J591, TLX591), developed in 1997, was evaluated in a phase 2 clinical trial in 2013 that evidenced a correlation between the injected dose and PSA decline, overall survival but also hematological toxicity [112]. A dose fractionation regimen was investigated and allowed higher cumulative dose, which resulted in improved efficacy in terms of PSA decline and survival, with comparable toxicity [113]. [^177^Lu]Lu-J591 was also tested in combination with docetaxel and appeared to be safe with early evidence of activity, 1 of the 15 patients achieving >50% PSA decline [114]. To date, numerous phase I/II clinical trials are underway with this radiobioconjugate radiolabeled with ^225^Ac (NCT03276572, NCT04506567, NCT04576871, NCT04886986, NCT04946370, NCT05567770) [115,116].

Less bulky than monoclonal antibodies, bombesin analog peptides are also interesting vectors for prostate cancer radionuclide therapy as they target a different protein from PSMA, namely the gastrin-releasing peptide receptor (GRPR). For example, [^177^Lu]Lu-RM2 has been studied in patients with metastatic castration-resistant prostate cancer, showing promising features for clinical use [117], and [^177^Lu]Lu-NeoBOMB1 is currently evaluated in a multicenter clinical trial (NCT03872778) in patients with advanced GRPR-overexpressing solid tumors.

A major challenge in the forthcoming years could consist in the efficient management of neuroendocrine-differentiated prostate adenocarcinoma [118], most often appearing secondary to treatment with AR inhibitors and displaying decreased or no expression of AR and PSMA [119]. In these diseases, PSMA targeting does not seem conclusive, as supported by PET imaging results [120,121,122,123]. However, several cases suggest that the use of SSTR-targeted radioligands could overcome this issue, including for PRRT purposes [124,125,126]. This encourages a precision oncology approach to prostate cancer management, considering the potential use of all available RLT options depending on the cellular expression of relevant molecular targets. In addition, alternative targets are being investigated for the radionuclide therapy of neuroendocrine PC, such as delta-like ligand 3 (DLL3) [127,128].

## 7. Conclusions

Radionuclide therapy is experiencing a new expansion phase. The increasing efforts to identify therapeutic targets that are specifically expressed on the surface of cancer cells potentially allow to focally deliver high energy to eradicate them. Radium-223 and ^177^Lu-labeled PSMA ligands have proven their benefit on the OS of mCRPC patients, with an acceptable safety profile. ^177^Lu-based RLT represents a further step towards precision medicine, also called personalized medicine, offering patients a treatment adapted to the characteristics of their tumor. Various radiopharmaceuticals are currently used or evaluated in prostate neoplasms, and numerous combination trials are underway, with the main objective of improving therapeutic response. As these treatments are currently available in the late stages of the disease, their indications will certainly evolve rapidly in the near future. Nevertheless, their use requires a perfect coordination between the nuclear scientists and the oncologists, as well as adapted infrastructures for efficient radioprotection.

## Figures and Tables

**Table 1:** Ongoing clinical trials with radionuclides for the treatment of prostate cancers (source: clinicaltrials.gov; last accession 15 November 2022). PCa: prostate cancer; BCR: biochemical recurrent; mHSPC: metastatic hormone sensitive prostate cancer; mCRPC: metastatic-castration resistant prostate cancer; ADT: androgen deprivation therapy; ARDT: androgen receptor directed therapy; EDTMP: ethylenediamine tetra(methylene phosphonic acid); EBRT: external beam radiation therapy; SBRT: stereotactic body radiation therapy; BAT: bipolar androgen therapy.

NCT Number	Drug Combined	Name of Study	Disease Stage	Intervention	Phase	Sponsor	Location
**NCT04206319**	-	20-C-0010	BCR PCa	**[** ^**223**^ **Ra]RaCl**_**2**_	2	National Cancer Institute	United States
**NCT04443062**	-	BULLSEYE	Oligo-mHSPC	**[** ^**177**^ **Lu]Lu-PSMA-617**	2	Advanced Accelerator Applications	Netherlands
**NCT04720157**	-	PSMAddition	mHSPC	**[**^**177**^**Lu]Lu-PSMA-617** + SoC vs. SoC alone (ARDT + ADT)	3	Novartis Pharmaceuticals	United States, Austria, Belgium, Canada, China, France, Germany, Japan, Netherlands, Poland, Singapore, Spain, Sweden, Switzerland, Taiwan, United Kingdom
**NCT05204927**	-	CURLu177PSM0001	mHSPC	**[**^**177**^**Lu]Lu-PSMA-I&T** vs. Abiraterone with prednisone or enzalutamide	3	Curium US LLC	United States
**NCT04663997**	-	PR21	mCRPC	**[**^**177**^**Lu]Lu-PSMA-617** vs. Docetaxel	2	Endocyte	Canada
**NCT03454750**	-	IRST185.03	mCRPC	**[** ^**177**^ **Lu]Lu-PSMA-617**	2	Istituto Scientifico Romagnolo per lo Studio e la cura dei Tumori	Italy
**NCT05219500**	-	TATCIST	mCRPC	**[** ^**225**^ **Ac]Ac-PSMA-I&T**	2	Excel Diagnostics and Nuclear Oncology Center	United States
**NCT05114746**	-	CAAA617A11201	mCRPC	**[** ^**177**^ **Lu]Lu-PSMA-617**	2	Novartis Pharmaceuticals	Japan
**NCT04647526**	-	SPLASH	mCRPC	**[**^**177**^**Lu]Lu-PSMA-I&T **after second-line hormonal treatment vs. Abiraterone or enzalutamide	3	POINT Biopharma	United States, Canada, France, Netherlands, Switzerland, United Kingdom
**NCT04689828**	-	PSMAfore	mCRPC	**[**^**177**^**Lu]Lu-PSMA-617** vs. ARDT	3	Novartis Pharmaceuticals	United States, Austria, Belgium, Canada, China, France, Germany, Japan, Netherlands, Poland, Singapore, Spain, Sweden, Switzerland, Taiwan, United Kingdom
**NCT03315260**	-	BAYER-19502	mCRPC	**[** ^**223**^ **Ra]RaCl**_**2**_	4	Bayer	Japan
**NCT04681144**	-	QOLRAD	mCRPC	**[** ^**223**^ **Ra]RaCl**_**2**_	4	Bayer	Colombia
**NCT04833517**	-	REALITY	mCRPC	^**177**^**Lu/**^**225**^**Ac/**^**161**^**Tb-PSMA ligand**, or ^**223**^**Ra**, or **[**^**153**^**Sm]Sm-EDTMP**, or ^**90**^**Y**-labeled microspheres for radioembolization	4	Universität des Saarlandes	Germany
**NCT04232761**	-	RAPIT	mCRPC	**[** ^**223**^ **Ra]RaCl**_**2**_	4	Bayer	Taiwan
**NCT04597125**	-	BAYER-20510	mCRPC	**[**^**223**^**Ra]RaCl**_**2**_ vs. 2nd line NAH therapy	4	Bayer	Australia, Austria, Czech Republic, Finland, France, Germany, Hong Kong, Hungary, Israel, Italy, Korea, Republic of Seoul, Lithuania, Poland, Russian Federation, Singapore, Spain, United Kingdom
**NCT03432949**	Dexamethasone	TRANCE	mCRPC	**[**^**223**^**Ra]RaCl**_**2**_ plus dexamethasone	4	Bayer	Canada
**NCT04037358**	SBRT	RAVENS	Oligo-mHSPC	SBRT vs. SBRT + **[**^**223**^**Ra]RaCl**_**2**_	2	Sidney Kimmel Comprehensive Cancer Center at Johns Hopkins	United States
**NCT05496959**	SBRT	LUNAR	Oligo-mHSPC	SBRT vs. SBRT + ^**177**^**Lu-PNT2002**	2	POINT Biopharma	United States
**NCT05230251**	EBRT	ROADSTER	PCa (local recurrence)	**[**^**177**^**Lu]Lu-PSMA-I&T** + High dose brachytherapy vs. High dose brachytherapy	2	London Health Sciences Foundation	London, Canada
**NCT03361735**	SBRT	NCI-2017-02192	mHSPC	ADT + SBRT + **[**^**223**^**Ra]RaCl**_**2**_	2	City of Hope Medical Center	United States
**NCT04343885**	CT	UpFrontPSMA	mHSPC	Sequential **[**^**177**^**Lu]Lu-PSMA-617** + Docetaxel vs. Docetaxel alone	2	Advanced Accelerator Applications	Australia
**NCT04704505**	BAT	BAT-RAD	mCRPC	**[**^**223**^**Ra]RaCl**_**2**_ + BAT	2	Sidney Kimmel Comprehensive Cancer Center at Johns Hopkins	Brazil
**NCT04419402**	Enzalutamide	ENZA-p	mCRPC	Enzalutamide ± **[**^**177**^**Lu]Lu-PSMA-617**	2	Endocyte Astellas Pharma Inc	Australia
**NCT02194842**	ARDT	PEACE III	mCRPC	Enzalutamide ± **[**^**223**^**Ra]RaCl**_**2**_	3	EORTC Bayer	Belgium, Brazil, Canada, Denmark, France, Ireland, Italy, Norway, Poland, Spain, Switzerland, United Kingdom
**NCT03574571**	CT	18-150	mCRPC	Docetaxel 75 mg/m^2^ vs. Docetaxel 60 mg/m^2^ + **[**^**223**^**Ra]RaCl**_**2**_	3	Bayer	United States, Netherlands
**NCT05150236**	IO	EVOLUTION	mCRPC	**[**^**177**^**Lu]Lu-PSMA-617** vs. **[**^**177**^**Lu]Lu-PSMA-617** + Ipilimumab + Nivolumab	2	Bristol-Myers Squibb Advanced Accelerator Applications	Australia

## Supplementary Materials

The following supporting information can be downloaded at: https://www.mdpi.com/article/10.3390/cancers15123133/s1, Table S1: Ongoing Phase 1 clinical trials with radionuclides for the treatment of prostate cancers.
