# Supplementary material for: Innovation in Radionuclide Therapy for the Treatment of Prostate Cancers: Radiochemical Perspective and Recent Therapeutic Practices
Source: Cancers (Basel). 2023 Jun 10;15(12):3133. doi: 10.3390/cancers15123133 (PMC10296341; doi:10.3390/cancers15123133)
Supplement: Supplementary file 1 [file cancers-15-03133-s001.zip › cancers-2368144-supplementary.pdf]

Supplementary Table S1: Ongoing Phase 1 clinical trials with radionuclides for the treatment of PCas (source: clinicaltrials.gov ; last accession : Novembre 15th 2022). PCa: Prostate Cancer, mHSPC: metastatic hormone sensitive Prostate Cancer, mCRPC: metastatic-castration resistant Prostate Cancer, ADT: androgen deprivation therapy, ARDT: Androgen receptor directed therapy, EBRT: External beam radiation therapy, SBRT: Stereotactic Body Radiation Therapy, BCR: biochemical reccurence, cN+: clinical positive lymph node.

NCT number	Drug combined	Name of Study	Disease stage	Intervention	Phase	Location
**NCT05079698**	SBRT	21-158	Oligo mHSPC	**^{177}Lu-PSMA-617** + Stereotactic Body Radiotherapy	Phase 1	United States
**NCT05162573**	EBRT	PROQURE-1	cN+ PCa	**^{177}Lu-PSMA-617 +** EBRT	Phase 1	Netherlands
**NCT04071236**	Avelumab Peposertib	NCI-2019-05620	mCRPC	**[^{223}Ra]$RaCl_2$** *vs* **[^{223}Ra]$RaCl_2$** + Peposertib *vs* **[^{223}Ra]$RaCl_2$** + Peposertib + Avelumab	Phase 1/2	United States
**NCT04597411**		AcTION	mHSPC mCRPC	**[^{225}Ac]Ac-PSMA-617**	Phase 1	South Africa
